# Evaluation of oxidative stress markers in pathogenesis of diabetic neuropathy

**DOI:** 10.1007/s11033-012-1722-9

**Published:** 2012-06-21

**Authors:** Jacek Kasznicki, Marcin Kosmalski, Agnieszka Sliwinska, Malgorzata Mrowicka, Malgorzata Stanczyk, Ireneusz Majsterek, Jozef Drzewoski

**Affiliations:** 1 Department of Internal Medicine, Diabetology and Clinical Pharmacology, Medical University of Lodz, ul. Parzeczewska 35, 95-100 Zgierz, Poland; 2 Department of Clinical Chemistry and Biochemistry, Medical University of Lodz, ul. PL. Hallera, 90-647 Lodz, Poland

**Keywords:** Diabetic neuropathy, Oxidative stress, Oxidative DNA damage, Activity of antioxidant enzymes, Nitric oxide, Antioxidant status

## Abstract

Experimental evidences suggest that hyperglycaemia-induced overproduction of reactive oxygen species and subsequent damage to proteins, lipids and DNA may play a key role in the development of distal symmetric polyneuropathy (DSPN)—the most common complication of diabetes mellitus. The study population consisted of 51 individuals aged 52–82 years classified into 3 groups: 16 patients diagnosed with type 2 diabetes mellitus (T2DM) with DSPN, 16 T2DM patients without DSPN and 19 control subjects without diabetes and neuropathy. The study was conducted to determine the activity of antioxidant enzymes: catalase (CAT), superoxide dismutase (SOD), glutathione peroxidase (GPX) and total antioxidant status (TAS) in the examined groups. An alkaline comet assay was used to determine the extent of DNA damage of oxidized purines as glicosylo-formamidoglicosylase (Fpg) sites, and oxidized pyrimidines as endonuclease III (Nth) sites. A significant decrease of SOD (*P* < 0.05), GPX (*P* < 0.05) and nonsignificant decrease of CAT (*P* > 0.05), and TAS status (*P* > 0.05) were seen in T2DM patients with neuropathy compared to T2DM patients as well as controls. T2DM patients with or without neuropathy revealed significantly lower (*P* < 0.05) plasma concentration of nitrous oxide compared to the control subjects. Endogenous level of oxidative DNA damage in T2DM patients with DSPN was significantly higher compared both to the controls and T2DM patients without DSPN (*P* < 0.001). Moreover, lymphocytes isolated from T2DM patients with DSPN were more susceptible to oxidative DNA lesions induced by hydrogen peroxide than from T2DM patients without DSPN (*P* < 0.001). Our results confirm hypothesis that oxidative stress may play a substantial role in the development and progression of diabetic distal symmetric polyneuropathy.

## Introduction

Diabetic neuropathies are the most common, and at the same time the least recognized and understood long-term complication of diabetes, occurring both in type 1 and type 2 diabetic patients [1–7].

It is estimated, that neuropathy may be present in up to 10 % of patients at diagnosis of type 2 diabetes mellitus (T2DM) and affects up to 50 % of patients with long term diabetes. In individual patients different parts of the nervous system may be involved and that is why diabetic neuropathies are heterogenous by clinical manifestation, course, and underlying mechanisms [1, 2, 7–9]. The most common form of this chronic complication of diabetes is distal symmetric polyneuropathy (DSPN). The diagnosis of DSPN is typically made in patients with poor glycemic control and its prevalence increases with age and duration of diabetes. This form of neuropathy is often accompanied by autonomic neuropathy [10]. Diabetic neuropathies exert strong, negative influence on both survival and quality of life [11, 12]. DSPN is the main cause of serious chronic complications of diabetes including foot ulceration, Charcot neuropathy, foot amputation, as well as numerous cardiovascular, gastrointestinal and/or genitourinary disorders [8, 10]. The early diagnosis and appropriate treatment of DSPN would be crucial to the prognosis of patients, because it could prevent or delay the development of numerous diabetic complications. However, no causative treatment is known since the pathogenesis of DSPN is not fully elucidated.

The etiology of DSPN seems to be heterogenous with several different factors being implicated [13]. Potential mechanisms leading to the functional and structural damage of nervous tissue include oxidative injury, activation of the polyol pathway of glucose metabolism, deposition of advanced glycosylation end products within the nerves and vascular insufficiency [13].

The purpose of this work was to assess the level of oxidative stress markers in patients with T2DM and DSPN compared to T2DM patients without DSPN as well as healthy subjects. DNA oxidative lesions were estimated by alkaline comet assay in lymphocytes from peripheral blood of T2DM patients with and without coexisting DSPN as well as healthy control subjects. We measured endogenous and exogenous DNA damage after hydrogen peroxide treatment (H_2_O_2_). An activity of antioxidant enzymes: catalase, superoxide dismutase and glutathione peroxidase as well as the total antioxidant status (TAS) were also estimated.

## Materials and methods

### Patients

The study population consisted of 51 unrelated Caucasians individuals residing in Lodz District, Poland. Subjects were enrolled into three groups, 16 T2DM patients with DSPN, 16 T2DM patients without clinical signs and symptoms of DSPN, and 19 apparently control subjects with normoglycemia without T2DM and clinical symptoms and signs of DSPN. Normoglycemia was defined as a fasting blood glucose <5.6 mmol/l and 2 h value <7.8 mmol/l. Patients were considered to have T2DM if the known diabetes was self-reported, diagnosis was included in their medical record and if they were taking medications for management of hyperglycemia. Diagnosis of T2DM was based on the American Diabetes Association definition of diabetes [14].

Exclusion criteria included lower limb amputation, psychiatric disorders, cancer or any genetic disease, terminal illness, evidence of peripheral arterial disease, claudication symptoms, alcohol abuse, thyroid disorders, vitamin B12 or folate deficiency, spondyloarthropathy, foot edema, hepatic disease, lumbosacral pathology, toxin exposure including chemotherapeutic agents, diagnosis of neuromuscular disorders, medical or surgical intervention for peripheral nerve pathology, inability to understand or provide inform consent. The control subjects had no known diagnosis of impaired glucose metabolism and neuropathy. All groups were matched for sex, age and diabetic groups were matched for the duration of type 2 diabetes. Characteristic of T2DM patients and controls is given in Table 1. All subjects were recruited from the Department of Internal Disease, Diabetology and Clinical Pharmacology between January 2009 and December 2010. The study was reviewed and approved by the institutional ethics committee of the Local Ethic Committee of the Medical University of Lodz and met the tenets of the Declaration of Helsinki. Written consent was obtained from each patient before enrolment in the study.

**Table 1:** Clinical and laboratory characteristics of type 2 diabetic patients (T2DM), type 2 diabetic patients with coexisting DSPN (T2DM + DSPN) and healthy control subjects (HS)

Parameter	T2DM	T2DM + DSPN	HS	*P*
	*n* = 16	*n* = 16	*n* = 19	
Sex (M/F)	6/10	8/8	10/9	NS
Age (years)	63.94 ± 11.83	65.69 ± 11.07	65.11 ± 14.47	NS
Weight (kg)	82.25 ± 18.08	81.68 ± 27.96	81.08 ± 25.27	NS
BMI (kg/m^2^)	30.47 ± 5.76	28.98 ± 10.35	28.77 ± 6.77	NS
A1C (%)	9.44 ± 1.68	9.15 ± 1.76	5.50 ± 0.31	<0.0001*
GFR (ml/min/1.72 m^2^)	87.13 ± 31.42	86.81 ± 49.30	84.42 ± 23.14	NS

Data are mean ± SD. *P* value < 0.05 is considered significant

*Denotes a statistically significant difference between control and both diabetic groups

### Diagnosis of DSPN

The diagnosis of DSPN was made based on presence of combination of symptoms (screening questionnaire) and signs of neuropathy including decreased distal sensation and/or decreased or absent ankle reflexes after elimination of confounding factors (inclusion/exclusion criteria) [7, 9, 15]. All the sensory measurements were performed by a single clinician in a patient in supine position.

### Symptoms of DSPN

A standardized questionnaire was completed to obtain demographic data, medical history including drugs and lifestyle factors. Symptoms of DSPN were determined from Michigan Neuropathy Screening Instrument [16].

### Signs of DSPN

Composite score was used to assess clinical signs using a modified neuropathy disability scores (NDS) comprising pinprick, vibration, temperature sensation, and Achilles reflexes [17, 18].

Vibratory perception was tested at the apex of the big toe with a 128 Hz graduated Rydel-Seiffer tuning fork [19, 20]. The test was conducted twice on each great toe. Patients were asked to close their eyes. Before examination, the sensation of vibration was demonstrated to the patient by applying the tuning fork to the wrist. The initial sham test was performed by applying non-vibrating tuning fork in order not to mistake the sensation of pressure for vibration.

Pin prick test was preformed proximally to big toenail. Temperature perception was performed on the dorsum of the foot with a tipterm. Pressure sensation was also assessed with Semmes–Weinstein 5.07 10 g monofilament in agreement with suggestions of American College of Foot and Ankle Surgeons [21]. Buckling of the monofilament was demonstrated first on the patient forearm. Patients were asked to close their eyes. Four sites on each foot were used in a random sequence—plantar surface of a great toe and plantar surface of the 1st, 3rd and 5th metatarsal head. One of the four applications on each foot was sham. Achilles tendon reflex was also determined in each patient.

## Blood sample preparation

Peripheral blood lymphocytes from blood of healthy donors and patients were isolated by centrifugation (15 min, 280 g) in a density gradient of histopaque-1077 (Sigma, Poznan, Poland). Lymphocytes accounted for about 92 % of leukocytes in the obtained cell suspensions as judged by the characteristic shape of their nucleus. Erythrocytes were separated from blood plasma by centrifugation (10 min, 710 g) at 4 °C and washed 3 times with 0.9 % NaCl before examination.

### Hemoglobin assay

Hemoglobin (Hb) concentration in erythrocytes hemolysate for enzyme activity determination was estimated at 540 nm using a spectrometer (UV/VIS Spectrometer Lambda 14P, Perkin Elmer, USA) after conversion into cyanmethemoglobin with Drabkin reagent (Aqua-Med, Poland) [22].

### Catalase activity

Catalase activity in erythrocytes was determined according to spectrophotometric procedure by Beers and Sizer [35] and calculated as Bergmeyer units (BU/g Hb). CAT activity was measured at 25 °C by recording H_2_O_2_ decomposition at 240 nm with a spectrometer (UV/VIS Spectrometer Lambda 14P, Perkin Elmer, USA). One Bergmeyer unit (BU) of CAT activity is defined as the amount of enzyme decomposing 1 g of H_2_O_2_ per min.

### Glutathione peroxidase activity

Glutathione peroxidase activity in erythrocytes was measured according to spectrophotometric procedure by Little and O’Brian [36] and presented as enzymatic units (U/g Hb). The difference in the rate of GPX reaction with glutathione and kumen in the sample was used for its activity determination by absorbance measurement with a spectrometer (UV/VIS Spectrometer Lambda 14P, Perkin Elmer, USA) at 412 nm. One unit of GPX activity is calculated as an amount of enzyme which causes 10 % decrease of the level of reduced glutathione within 1 min at 25 °C, pH 7.0.

### Superoxide dismutase activity

Superoxide dismutase activity in erythrocytes was measured according to procedure by Misra and Ridovich [23] and expressed in adrenaline units (U/g Hb/100 ml). The activity was determined at 37 °C by the absorbance increase at 480 nm with a spectrometer (UV/VIS Spectrometer Lambda 14P, Perkin Elmer, USA) following by the auto-oxidation of adrenaline inhibited by SOD. One unit of SOD activity is defined as the amount of enzyme inhibiting the adrenaline autooxidation at 50 %.

### Total antioxidant status determination

Determination of the total antioxidant status in blood plasma was performed by spectrophotometric method according to procedure no. NX2332 by Randox (Randox Laboratories Ltd., Ardmore, Diamond Road, Crumlin, Co Antrim, United Kingdom, BT29 4QY). Briefly, ABTS [2,2′-Azino-di-(3-ethylbenzthiazoline sulphonate)] was incubated with peroxide (metmyoglobin) and H_2_O_2_ produce the radical cation ABTS with a relatively stable blue-green color. Antioxidants when added to examined sample caused suppression of this color production measured as decrease of absorbance with a spectrometer (UV/VIS Spectrometer Lambda 14P, Perkin Elmer, USA) at 600 nm. The total antioxidant status was calculated as concentration of antioxidants (mmol/l).

### Comet assay

DNA damage of single- and double-strand breaks levels was measured by the single-cell electrophoresis method. The final concentration of lymphocytes was adjusted to 1–3 × 105 cells/ml by adding RPMI-1640 medium (Sigma, Munich, Germany) to the single cell suspension. Endogenous and exogenous DNA damage after lymphocyte incubation for 10 min at 4 °C with 10 μM hydrogen peroxide at 4 °C in growth medium was investigated. The comet assay was performed under alkaline conditions according to the procedure of Singh, with modifications by Klaude [24, 25]. A suspension of cells in 0.75 % low melting point (LMP) agarose dissolved in PBS was spread onto microscope slides (Superior Marienfeld, Lauda-Königshofen, Germany) precoated with 0.5 % normal-melting agarose. The cells were then lysed for 1 h at 4 °C in a buffer consisting of 2.5 M NaCl, 100 mM EDTA, 1 % Triton X-100, and 10 mM Tris, pH 10. After lysis, the slides were placed in an electrophoresis unit, and the DNA was allowed to unwind for 40 min in the electrophoretic solution consisting of 300 mM NaOH and 1 mM EDTA, pHN13. Electrophoresis was conducted at 4 °C (the temperature of the running buffer did not exceed 12 °C) for 30 min at the electric field strength of 0.73 V/cm (30 mA). The slides were then neutralized with 0.4 M Tris, pH 7.5, stained with 2 μg/ml DAPI, and covered with cover slips. To prevent additional DNA damage, all the steps described previously were conducted under dimmed light or in the dark.

### Endonuclease assay

Fpg and Nth nicks oxidized DNA, giving breaks that can be detected by the alkaline comet assay. Nth converts oxidized pyrimidines into strand breaks [26]. Fpg is involved in the first step of the base excision repair to remove specific modified bases from DNA to create an apurinic or an apyrimidinic site (AP-site), which is subsequently elevated by its AP-lyase activity giving a gap in the DNA strand [27]. The gap can be detected by the comet assay. The enzyme excises mainly 2,6-diamino-4-hydroxy-5-*N*-methyl formamidopyrimidine [28] and 7,8-dihydro-8-oxo-2′deoxyguanine (8-oxo-G) [26, 29]. Endogenous and exogenous oxidative DNA lesions after lymphocyte incubation for 10 min at 4 °C with 10 μM hydrogen peroxide at 4 °C in growth medium were investigated. According to the standard method of comet assay, slides after lysis were washed three times in an Fpg/NthI buffer comprising 40 mM HEPESKOH, 0.1 mM KCl, 0.5 mM EDTA, 0.2 mg/ml bovine serum albumin, pH 8.0, and the agarose was covered with 25 ml of buffer or NthI as well as Fpg at 1 mg/ml in buffer, sealed with a cover glass, and incubated for 30 min at 37 °C. Further steps were made as described previously.

### Comet analysis

The objects were observed at 200× magnification in an Eclipse fluorescence microscope (Nikon, Tokyo, Japan) attached to a COHU 4910 video camera (Cohu, San Diego, CA) equipped with a UV-1 filter block (an excitation filter of 359 nm and a barrier filter of 461 nm) and connected to a personal computer-based image analysis system Lucia-Comet v. 4.51 (Laboratory Imaging, Prague, Czech Republic). Two parallel tests with aliquots of the same sample of cells were performed.

### Statistical analysis

The data point in this study was calculated for three separate experiments from each analyzed patient or control sample. The value from comet assay was expressed as mean percentage of DNA damage ± SEM. The activity of enzymes as well as the total antioxidant status was expressed as mean value ± SD. Blinded replicate samples were used for quality control (QC). If no significant differences between variations were found, as assessed by the Snedecor–Fisher test, the differences between means were evaluated by applying the Student’s *t* test. Otherwise, the Cochran–Cox test was used. The data were analyzed using the STATISTICA (StatSoft, Tulsa, OK) statistical package.

## Results

### Total antioxidant status

**Fig. 1:**
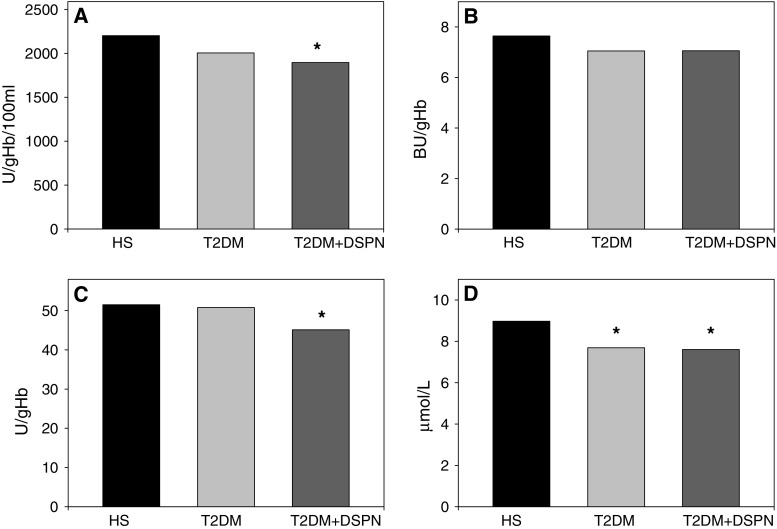
Mean activity of superoxide dismutase (SOD) calculated in adrenaline units (U/g Hb/100 ml) (**a**), catalase (CAT) calculated in Bergmeyer units (BU/g Hb) (**b**), glutathione peroxidase calculated in enzymatic units (U/g Hb) (**c**) and nitric oxide concentration calculated as (μmol/l) (**d**) measured in T2DM patients with DSPN (T2DM + DSPN), T2DM patients without DSPN (T2DM) and healthy subjects (HS). Each *data point* represents the mean ± SD. * *P* < 0.05 as compared with healthy subjects

**Fig. 2:**
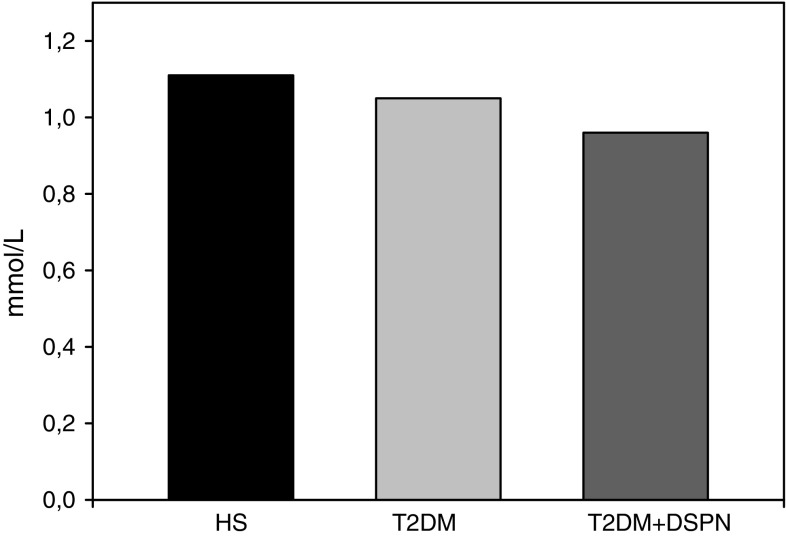
Total antioxidant status (TAS) in T2DM patients with DSPN (T2DM + DSPN), T2DM patients without DSPN (T2DM) and healthy subjects (HS) calculated as concentration of antioxidants (mmol/l). Each *data point* represents the mean ± SD. * *P* < 0.05 as compared with healthy subjects. ^#^ *P* < 0.05 comparison between T2DM patients and T2DM patients with DSPN

It was found that the erythrocyte activity of SOD (U/gHb/100 ml) was significantly decreased in T2DM patients with DSPN compared to healthy subject (1,895 ± 193 vs. 2,201 ± 529; *P* < 0.05). T2DM patients with DSPN revealed no significantly lower activity as compared to T2DM patients (1,895 ± 193 vs. 2,005 ± 320; *P* > 0.05) (Fig. 1a). The CAT activity (BU/gHb) was lower in T2DM patients with and without DSPN in comparison to controls, but differences did not reach a significant statistical power (7.06 ± 0.9 vs. 7.05 ± 1.2 vs. 7.6 ± 1.3; *P* > 0.05) (Fig. 1b). The activity of GPX (U/gHb) was markedly diminished in T2DM patients with coexisting DSPN compared to healthy subject (45.1 ± 8.8 vs. 51.5 ± 9.2; *P* < 0.05). The GPX activity in the examined group of patients with DSPN was lower, but non-statistically different compared to T2DM patients without DSPN (45.1 ± 8.8 vs. 50.8 ± 8.9; *P* > 0.05) (Fig. 1c). The NO level (μmol/L) was significantly decreased in T2DM patients with and without DSPN in comparison to healthy controls (7.6 ± 0.9 vs. 7.7 ± 1.3 vs. 8.9 ± 1.6; *P* < 0.05) (Fig. 1d). The plasma concentration of TAS (mmol/L) showed no significantly lower level in T2DM patients with DSPN compared to T2DM patients and healthy subject (0.96 ± 0.3 vs. 1.05 ± 0.2 vs. 1.11 ± 0.3; *P* > 0.05) (Fig. 2).

### Oxidative DNA damage

**Fig. 3:**
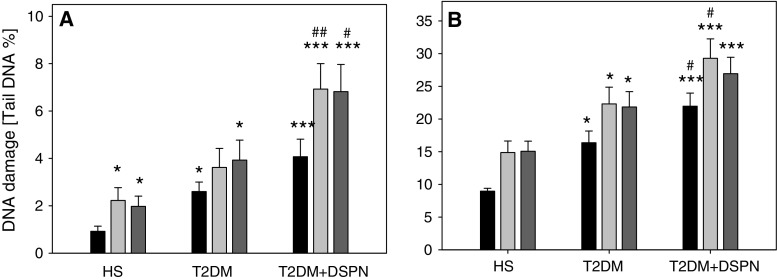
The level of endogenous (**a**) and H_2_O_2_-induced (**b**) DNA damage in lymphocytes of T2DM with DSPN (T2DM + DSPN), T2DM patients without DSPN (T2DM) and healthy subjects (HS). The cells were treated or not with 10 μM H_2_O_2_ for 10 min at 4 °C with subsequent treatment with endonuclease III (Nth) or formamidopyrimidine-DNA glycosylase (Fpg) at 1 μg/mL. The *black bars* (control) present DNA strand breaks and alkaline labile sites, the *light grey bars* (Fpg) present oxidized purines and the *dark grey bars* (Nth) present oxidized pyrimidines. The DNA damage was measured as the percentage of tail DNA in the alkaline comet assay. Each *data point* represents the mean ± SEM.*** *P* < 0.001, ** *P* < 0.01, * *P* < 0.05 as compared with healthy subjects. ^#^ *P* < 0.05, ^##^ *P* < 0.01, ^###^ *P* < 0.05 comparison between T2DM patients and T2DM diabetic patients with DSPN

The mean level of endogenous and oxidative DNA damage was significantly higher in lymphocytes of T2DM patients without DSPN than in control group (*P* = 0.001). Lymphocytes of T2DM patients with DSPN showed a trend towards a higher level of endogenous and oxidative DNA damage than lymphocytes of T2DM patients without DSPN (*P* = 0.092). Endogenous DNA damages seem to result from the action of endogenous DNA damaging agents which affect the cells e.g., reactive oxygen species produced during aerobic metabolism or action of DNA repair proteins. Oxidative DNA damage revealed after treatment with endonucleases Fpg and Nth were higher in lymphocytes of T2DM patients without DSPN (Fpg *P* = 0.147; Nth *P* = 0.039) and T2DM patients with DSPN (Nth *P* < 0.001; Nth *P* < 0.001) compared to endogenous DNA damage in control subjects (Fig. 3a).

The mean level of hydrogen peroxide-induced DNA damage was markedly higher in lymphocytes of T2DM patients than in control subjects (*P* < 0.001). Moreover, the mean level of hydrogen peroxide-induced DNA damage in lymphocytes of T2DM patients with DSPN was significantly higher than in T2DM patients without DSPN (*P* = 0.045). Oxidative DNA damage evoked by hydrogen peroxide revealed after treatment with endonucleases Fpg and Nth were significantly higher in lymphocytes of T2DM patients without DSPN (Fpg *P* = 0.019; Nth *P* = 0.018) and T2DM patients with DSPN (Fpg *P* < 0.001; Nth *P* < 0.001) than in control subjects. Lymphocytes of T2DM patients with DSPN showed a trend towards a higher level of oxidative DNA damage induced by hydrogen peroxide (revealed after treatment with endonucleases Fpg and Nth) as compared to lymphocytes of T2DM patients alone (Fpg *P* = 0.06; Nth *P* = 0.15) (Fig. 3b).

## Discussion

Although, no theory is completely accepted as being the single cause of neuropathy, it is regarded that reactive oxygen species (ROS) generated in vivo play an important role in nerve damage [13, 30]. In diabetic patients, poor glycemic control leads to chronic hyperglycemia. The oxidation of elevated levels of glucose within the cell stimulates production of ROS and increases oxidative stress [30]. An increased generation of ROS such as superoxide, hydrogen peroxide and hydroxyl radical is the cause of oxidation and modification of structure of cellular proteins, nucleic acids, and membrane lipids. Higher levels of peroxynitrate were found by Al-Nimer et al. [31] in biological fluids of T2DM patients compared to healthy subjects. The difference was highly significant for T2DM subjects with coexisting DSPN. Damage of the cellular structure and impairment of its function, leads to cell necrosis and activation of genes involved in neuronal damage [32]. In diabetic patients increased oxidative stress may be associated not only with an overproduction of ROS, but also with a significant decrease in the effectiveness of antioxidant defenses or both. Insufficient anti-oxidative cellular mechanisms may also be involved in nerve damage. Its seems that the activity of cellular antioxidants such as the enzymes superoxide dismutase (SOD), catalase (CAT), and glutathione peroxidase (GPX) may be crucial to this process.

In our study, a decreased activity of primary antioxidant enzymes such as catalase, superoxide dismutase and glutathione peroxidase in peripheral blood of T2DM patients with coexisting DSPN has been demonstrated. Superoxide dismutase (EC 1.15.1.1) catalyzes the oxidation/reduction conversion of superoxide radicals to molecular oxygen and hydrogen peroxide. The protein has been known for over 30 years as a copper-containing, low molecular weight cytoplasmic protein identified in erythrocytes [33, 34]. Catalase (EC 1.11.1.6) is a second key antioxidant enzyme involved in a protection against harmful peroxide [35]. Catalase is a heme enzyme that converts hydrogen peroxide to water and oxygen and thereby mitigates its toxic effects. Selenoprotein glutathione peroxidase (EC 1.11.1.9) catalyzes the hydrogen peroxide reduction by two molecules of glutathione (GSH) as a part of reactive oxygen species defense system [35, 36]. Besides hydrogen peroxide, GSH is involved in a large number of intracellular detoxifications [36]. We found a significant decrease of the activity of SOD, GPX and CAT in T2DM patients with DSPN. Interestingly, El-Boghdady and Badr [37] found a significant decrease of erythrocyte GSH in diabetic patients with and without DSPN compared to healthy subjects.

According to SOD, GPX and CAT we also estimated the level of the total antioxidant status in blood samples of T2DM patients as well as control subjects. TAS status may result directly from abnormalities of antioxidant enzymes and/or natural scavengers including glutathione, carotenoids, ubihydrochinon, tocopherols, vitamins A, C, E etc., which support the antioxidant barrier of the human body. Finally, the measurement of the total antioxidant status revealed insignificant decrease of TAS in T2DM patients with coexisting DSPN enrolled in our study. This observation is not supported by all investigators, since El-Boghdady and Badr [37] found an increase in total antioxidant status in T2DM patients with coexisting DSPN.

We also evaluated plasma nitric oxide (NO) concentration in the study groups. There were significant differences between T2DM patients with and without DSPN compared to healthy controls. Our results showed that chronic high glucose concentration induced the decrease of plasma NO level in diabetic patients. It was reported that simulated hyperglycemia resulted in a significant down-regulation of eNOS expression and NO production by cultured human coronary endothelial cells [38, 39]. However, several earlier studies have demonstrated increased NO production in diabetes [40–42].

Endothelial NO, along with prostacyclin I2 (PGI2), is the main vasodilator and the most important factor involved in the function of blood vessels. Endothelial dysfunction is characterized by low bioavailability of endothelium-derived NO. It seems that reduced antioxidative defense system in diabetic patients is associated with increased vascular oxidative stress through decreased NO bioavailability. NO is inactivated by the superoxide radical and the peroxynitrite anion that can cause endothelial damage. Moreover, free radicals, especially superoxide anion may react with endothelium-derived nitric oxide (NO) and can inhibit endothelial nitric oxide synthase (eNOS). This interaction is one of the most important mechanisms involved in the endothelial dysfunction in diabetic patients [43]. ROS has been reported to contribute to impartment of endothelium-dependent vascular relaxation by the inactivation of NO, and generally to the vascular dysfunction resulting in accelerated atherosclerosis in diabetic patients. It is suggested that an increased production of ROS, induced by hyperglycemia, is involved in platelet dysfunction, tissue remodeling (via metalloproteinases), and glucose transport in skeletal muscle [44].

It is speculated, that the hyperglycemia-induced augmented oxidative stress contributes to the development of diabetic microangiopathies, including neuropathy. The oxidative damage to the macromolecules (lipid, protein, DNA) is well recognized in T2DM patients with microvascular complications [45, 46]. It is also documented that diabetic patients with and without microangiopathies have increased level of oxidative DNA damage [47–49]. Growing evidence suggest that one of causes of increased level of oxidative DNA damage in diabetic patients is reduced antioxidant defense system [50]. It was demonstrated that in T2DM primary antioxidant enzymes such as superoxide dismutase, catalase, and glutathione peroxidase have an altered activity [51]. These abnormalities may result in an increased risk of chronic diabetes complications, including neuropathy. Our results confirmed these findings and we observed lower activity of antioxidant enzymes as well as total antioxidant status of T2DM patients with coexisting DSPN. Moreover, Merzouk et al. [52] have shown that T2DM patients have significantly lower levels of antioxidative vitamins A and E. Therefore, we also investigated an association between total antioxidant status and the level of oxidative DNA damage which can be evaluated as a marker of oxidative stress in patients with DSPN.

Our results revealed that lymphocytes of T2DM patients with and without DSPN had significantly higher level of basal and oxidative DNA damage. These results stay in agreement with previous data [53, 54]. We also found that lymphocytes of T2DM patients with and without DSPN were more susceptible to DNA damage induced by hydrogen peroxide. This observation may be a result of insufficient antioxidant protection in diabetic patients and decreased level of the endogenous and exogenous free radicals scavengers [55–57]. The present work provides evidences that the risk of the development of DSPN may be associated with oxidative stress, since we observed significantly increased level of oxidative DNA damage in lymphocytes of T2DM patients with coexisting DSPN as compared to T2DM patients without DSPN as well as control subjects. The patients group in our study, displayed enhanced sensitivity to hydrogen peroxide treatment compared to controls as estimated by the level of oxidative DNA lesions. In our ongoing research we also investigate gene polymorphisms of antioxidant enzymes (the −262 C/T CAT, the Pro198Leu GPX and the 35 A/C SOD polymorphisms) as well as a key factors of DNA base excision repair (the Arg399Glu XRCC1, Tyr165Cys MUTYH, Ser326Cys OGG1 polymorphisms) and we can assume that some of them, especially SOD, GPX and OGG1 genes may be associated with increased risk of DSPN occurrence in Polish population (data not shown). Recent studies confirmed our findings, suggesting an important role of genetic factors in pathogenesis of diabetic neuropathy associated with oxidative stress [58]. Hovnik et al. [58] investigated the 116Val/Ala SOD, the -262C/T CAT gene polymorphisms, glutathione-S-transferases GSTM1 and GSTT1 polymorphic deletions as well as number of pentanucleotide (CCTTT)n repeats in inducible nitric oxide synthase in patients group with type 1 diabetes. Finally, he reported that selected polymorphisms of investigated genes could be added to a panel of genetic markers for identification of individuals at an increased risk for developing diabetic retinopathy.

Oxidative stress is hypothesized to play a role in the development of many neuronal-related chronic or late-onset diseases including Alzheimer’s disease, Parkinson’s disease, amyotrophic lateral sclerosis, Huntington’s disease, glaucoma or diabetic retinopathy [59–61]. The abnormalities of the pro-oxidant/antioxidant status can lead to oxidative damage, especially when the enzymatic defense system weakens in the elderly. In our previously reported study, we demonstrated that reactive oxygen species might promote localized DNA damage in glaucoma-iris tissues of elderly patients vulnerable to diabetic injury [62]. Chronic changes in the composition of antioxidants present in diabetic patients may induce alterations in neuronal cells. An overproduction of oxidants as a consequence of a decrease in antioxidant defenses and/or impaired DNA repair is considered to cause large extent of oxidative damage to lipids, proteins and DNA resulting in apoptosis induction [63]. It is suggested that vascular damage and hypoxia, often associated with T2DM, might lead to apoptosis of nerve ganglion cells and might also contribute to the induction of oxidative damage in the T2DM patients. These findings implicate that ROS play a fundamental role during the development of diabetic neuropathy. Moreover, our data suggests a large impairment of antioxidant defense system in T2DM patients. In conclusion, the present study supports the hypothesis that oxidative stress is an important factor in the pathogenesis of DSPN, therefore it may be a relevant target for the prevention and therapy of this complication of DM.
